# Protective effect of *Malva sylvestris L*. extract in ischemia-reperfusion induced acute kidney and remote liver injury

**DOI:** 10.1371/journal.pone.0188270

**Published:** 2017-11-20

**Authors:** Houshang Najafi, Zeynab Mohamadi Yarijani, Saeed Changizi-Ashtiyani, Kamran Mansouri, Masoud Modarresi, Seyed Hamid Madani, Bahar Bastani

**Affiliations:** 1 Medical Biology Research Center, Kermanshah University of Medical Sciences, Kermanshah, Iran; 2 Department of Physiology, School of Paramedical Sciences, Arak University of Medical Sciences, Arak, Iran; 3 Pharmacognosy and Biotechnology Department, Faculty of Pharmacy, Kermanshah University of Medical Sciences, Kermanshah, Iran; 4 Department of Pathology, School of Medicine, Kermanshah University of Medical Sciences, Kermanshah, Iran; 5 Division of Nephrology, School of Medicine, Saint Louis University, Saint Louis, Missouri, United States of America; University of PECS Medical School, HUNGARY

## Abstract

Mallow (*Malva sylvestris L*.) has had medicinal and therapeutic uses in addition to its oral consumption. The present study was conducted to examine the protective effect of *Malva sylvestris L*. extract on ischemia-reperfusion-induced kidney injury and remote organ injuries in the liver. Before ischemia-reperfusion, rats in the different groups received intraperitoneal normal saline or mallow extract at the doses of 200, 400 or 600 mg/kg of body weight. After 30-minutes of bilateral renal ischemia followed by 24-hours of reperfusion, tissue damage in the kidney and liver samples were determined through studying H&E-stained slides under a light microscope. The degree of leukocyte infiltration and tissue mRNA expressions of TNF- and ICAM-1 were then measured to examine the degree of renal inflammation. The renal tissue MDA and FRAP levels were measured for determining the amount of oxidative stress. Plasma concentrations of creatinine, urea, ALT and ALP were also measured. Ischemia-reperfusion led to a significant increase in plasma concentrations of creatinine, urea, ALT and ALP, and renal tissue MDA, and a significant decrease in renal tissue FRAP. The expression of pro-inflammatory factors in the kidney tissue, the level of leukocyte infiltration and the amount of tissue damage in the kidney and liver also increased. Pretreatment by mallow extract led to a significant improvement in all the variables measured. The 200- and 400-mg doses yielded better results in most parameters compared to the 600-mg dose. The findings showed that mallow extract protects the kidney against ischemia-reperfusion and reduces remote organ injury in the liver.

## Introduction

Mallow (Malva sylvestris L.) is an annual herb from the Malvaceae family that grows in various parts of the world, including south Europe, North Africa and southwest Asia [1]. In addition to being orally consumed, mallow has been used for medicinal and therapeutic purposes since 3000 BC due to its laxative, emollient and anti-inflammatory properties [2].

Phytochemical studies on mallow have shown that its various parts contain flavonoids [3–4], terpenoids [5], phenol derivatives [3, 5], polysaccharides [6], mucilages and coumarins [7], vitamins C and E and beta-carotene [3], fatty acids and various sterols, particularly essential fatty acids such as omega-3 and omega-6 [3, 8], chemical elements [9], enzymes such as sulfite oxidase and catalase [10–12] and amino acids [13,14].

Many studies have examined mallow and proposed numerous properties for this herb. Extracts from mallow leaves show anti-complementary properties [15], suppress release of pro-inflammatory mediators PGE2 and PGD2 [16], and have anti-inflammatory properties [17–21].

Mallow extract also shows antioxidant properties [3, 6, 18], and can destroy H2O2 due to its catalase activity [12]. Mallow extract is also shown to protect against the hepatotoxicity caused by paracetamol [22], and to protect the kidney against vanadium-induced damage [23].

Acute kidney injury (AKI) is a serious complication with no special treatments at the moment. Renal ischemia-reperfusion (IR) is one of the important causes of AKI. The pathophysiology of IR-induced renal disorders involves inflammation, oxidative stress and damage to the vascular endothelium and tubule epithelium [24–25]. Furthermore, AKI can cause remote organ injuries involving liver, lung, heart, spleen and brain tissues [25]. Gardner et al. demonstrated that a 40-minute renal ischemia followed by 24-hours of reperfusion increased liver enzymes in pigs [26]. Interestingly, melatonin reduced hepatic injuries caused by renal IR [27].

The present study was conducted to examine the protective effects of mallow hydro-alcoholic extract against the renal injuries caused by ischemia-reperfusion and its consequent remote liver injury in rats.

## Materials and methods

This research was designed and carried out based on the European Economic Community Guidelines for the care and use of laboratory animals (EEC Directive of 1986; 86/609/EEC) and was approved by the Ethics Committee of Kermanshah University of Medical Sciences (KUMS.REC.1394.85). Attempts were made to put the minimum possible number of animals in each group. If, during the experiment, any of the animals showed signs of unexpected pain and suffering (e.g. disability, reduced mobility and an abnormal state), they were removed from the study and euthanized by deep anesthesia.

### Extract preparation

The hydro-ethanol extract of mallow leaves was used in this study by purchasing fresh mallow flowers from a local herb shop in Kermanshah, Iran, and drying them in the shade. The flowers were approved by herb experts at this university’s Faculty of Pharmacy and samples of the herb were kept at the faculty’s herbarium. Extraction was performed at the Herb Lab of this faculty.

One liter of 70% ethanol was added to 60 grams of dried mallow flower and kept for 24 hours in a dark place inside a shaker. After filtering, the liquid phase was evaporated in vacuum using a rotary evaporator at 40°C and was stored in the dark at -20°C until use [28, 29].

### Experimental procedures and animal study

This study was conducted on 35 male Wistar rats weighing 250–300 grams (10–12 weeks old), taken from the Animal Breeding Center of Kermanshah University of Medical Sciences. The animals were housed in a room with a 12-hour light-dark cycle at 23±2°C and 55% humidity in cages (three rats per cage during acclimatization period, but after starting to test each rat in a separate cage). The rats had access to food and water ad libitum throughout the entire experiment. The rats were randomly divided into five groups (n = 7), including the sham control group, which received normal saline and underwent a sham surgery, the IR (Ischemia-Reperfusion) group, which received normal saline and 30-minutes of bilateral renal ischemia, and three IR+M groups, which received mallow extract at doses 200, 400 and 600 mg/kg of BW half an hour before ischemia was induced with a protocol similar to the protocol that was used for administering normal saline to the IR group. These doses were chosen based on the results of similar studies conducted in the past [22]. After the ischemia induction ended, rats were returned to their cages for 24-hours (reperfusion period) during which they had access to food and water ad libitum [30]. To induce anesthesia, 50 mg/kg of pentobarbital sodium was used, and half an hour before the ischemia, normal saline was injected to the sham and IR groups and the various doses of the extract were then administered intraperitoneally (ip) to the IR+M groups. Thirty minutes prior to the induction of anesthesia, all the animals received 50 international units (IU) of heparin. Throughout surgery, their body temperature was measured with a rectal probe (Physitemp BAT-12) and maintained at 37±1°C using a heat lamp and heat plate [30]. Once the 24-hour reperfusion period ended, the animals were anesthetized again and blood samples were taken from their abdominal aorta so as to measure their plasma concentrations of creatinine, urea, ALT and ALP.

Their left kidney was then removed and one half of it was stained with H&E for histopathological examination under a light microscope. The cortical tissue of the other half was also separated and was immediately frozen in liquid nitrogen for measuring the mRNA expression of TNF-α and ICAM-1. The right kidney was transferred to a -70°C freezer after rapid freezing in liquid nitrogen for examining oxidative stress through measuring malondialdehyde (MDA) and ferric reducing/antioxidant power (FRAP). A sample of the liver tissue was collected, processed and H&E stained to be examined under an optical microscope for possible tissue damage. Once the experiment ended, the rats were euthanized by injection of an overdose (100 mg/kg) of pentobarbital sodium anaesthetic.

### Assessing kidney and liver dysfunction

To examine kidney function, plasma concentrations of creatinine and urea were measured in the rats, and their liver function was also assessed by measuring their ALT and ALP levels.

### Assessing kidney and liver tissue damage

To examine kidney and liver tissue damage, a pathologist studied the H&E-stained tissue sections. Kidney tissue damage was investigated and graded for increased Bowman's space, intracellular vacuolization, necrosis of the tubular cells, vascular congestion and intratubular cast in ten microscopic fields [30–31]. The liver slides were also used to examine the level of leukocyte infiltration, cellular necrosis and congestion, and these damages were graded as poor, moderate and severe.

### Assessing inflammation in the kidney tissue

The mRNA expression of the pro-inflammatory factors TNF-α and ICAM-1 and the level of leukocyte infiltration into the interstitial space were measured to assess inflammation. The mRNA expression of TNF-α and ICAM-1 was measured by reverse transcription-polymerase chain reaction (RT-PCR) using beta-actin as the housekeeping gene in the renal cortical tissue. Table 1 presents the sequence of the primers used for each of the genes. The PCR products were also run on 2% agarose gel [29]. To examine the level of leukocyte infiltration, they were counted in 20 microscopic fields (each field area was 0.14 mm^2^) and the average count was calculated in each Mm^2^ [30].

**Table 1:** Primer sequences used to amplify mRNAs encoding proinflammatory cytokines.

Primer	Sequence		Annealing t°C	Product size (bp)
TNF-α	F	GAAAGGACACCATGAGCACG	58	264
	R	GAGAAGATGATCTGAGTGTGAGG		
ICAM-1	F	CAGCAGACCACTGTGCTTTGA	61	406
	R	GTCGAGCTTCAGGACCCTAGT		
β-Actin	F	GCCATGTACGTAGCCATCCA	60	375
	R	GAACCGCTCATTGCCGATAG		

TNF-α: Tumor necrosis factor-alpha; ICAM-1: Intercellular adhesion molecule-1.

### Assessing oxidative stress in the kidney tissue

To examine oxidative stress, MDA level was measured as the final product of the membrane lipid peroxidation and FRAP as indicative of the total antioxidant activity of the tissue using colorimetric assay [29, 32].

### Statistical analysis

SPSS-16 was used to statistically analyze the data, which were presented as mean±SEM. The one-way ANOVA and Duncan's post-hoc test were used to compare the data pertaining to the kidney and liver functions, oxidative stress and leukocyte infiltration in the kidney. The exact level of statistical significance (P) was determined using the LSD test. Non-parametric tests including the Kruskal-Wallis test and Mann-Whitney’s U-test were used to compare the values obtained for histopathological damage. The level of statistical significance was set at P<0.05.

## Results

### The effect of mallow extract on renal IR-induced renal and hepatic dysfunction

**Fig 1:**
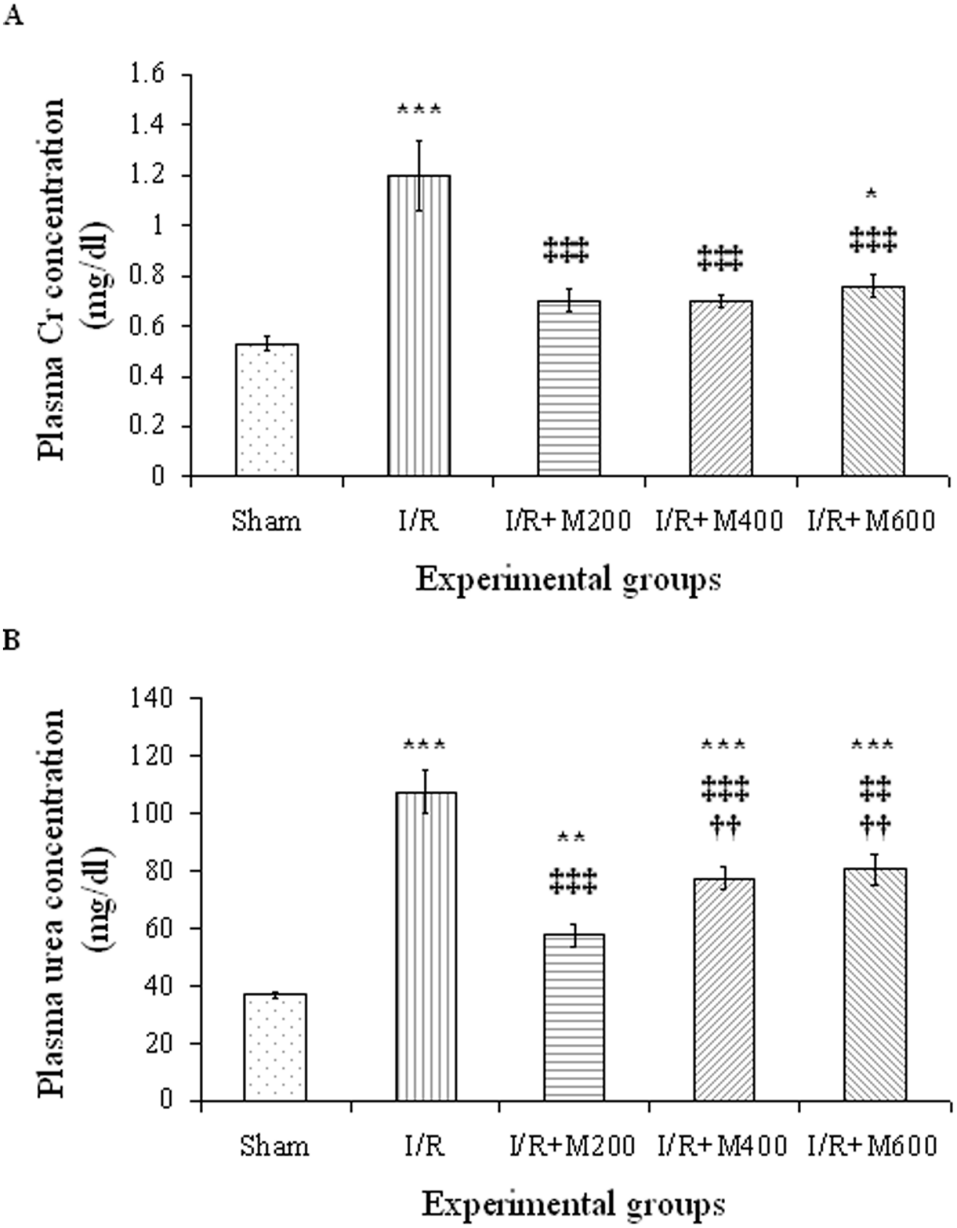
Plasma creatinine (A) and urea (B) levels in rats that underwent renal ischemia/reperfusion (I/R), pretreated with normal saline (I/R), or malva silvestris extract at 200, 400, or 600 mg/kg (I/R + M) compared to the sham group. Data is presented as mean ± SE (n = 7 in each group). * P<0.05 in comparison with the sham group ** P<0.01, *** P<0.001 in comparison with the sham group ‡‡ P < 0.01, ‡‡‡ P < 0.001 in comparison with the I/R group †† P < 0.01 in comparison with the I/R + M200 group.

Fig 1A and 1B show that 30-minutes renal ischemia followed by 24-hours of reperfusion caused a significant rise in plasma concentrations of creatinine (126%) and urea (189%) in the IR group compared to the sham group (P<0.001 for both). All three doses of mallow extract significantly reduced plasma creatinine concentrations (P<0.001 for all three doses vs I/R group), to levels similar to that in the sham group in both the IR+M200 and IR+M400 groups (Fig 1A). In the IR+M600 group, however, it had a significant difference with the sham group (P<0.05).

Plasma concentrations of urea was also significantly reduced the in all three groups receiving mallow extract, although its effect was the highest in the IR+M200 group (Fig 1B).

Plasma concentrations of urea in the three groups receiving mallow extract were still significantly higher than the value in the sham group (P<0.01 for the IR+M200 group, and P<0.001 for the IR+M400 and IR+M600 groups). It is worth noting that plasma urea concentrations in the IR+M400 and IR+M600 groups were significantly higher than in IR+M200 group.

**Fig 2:**
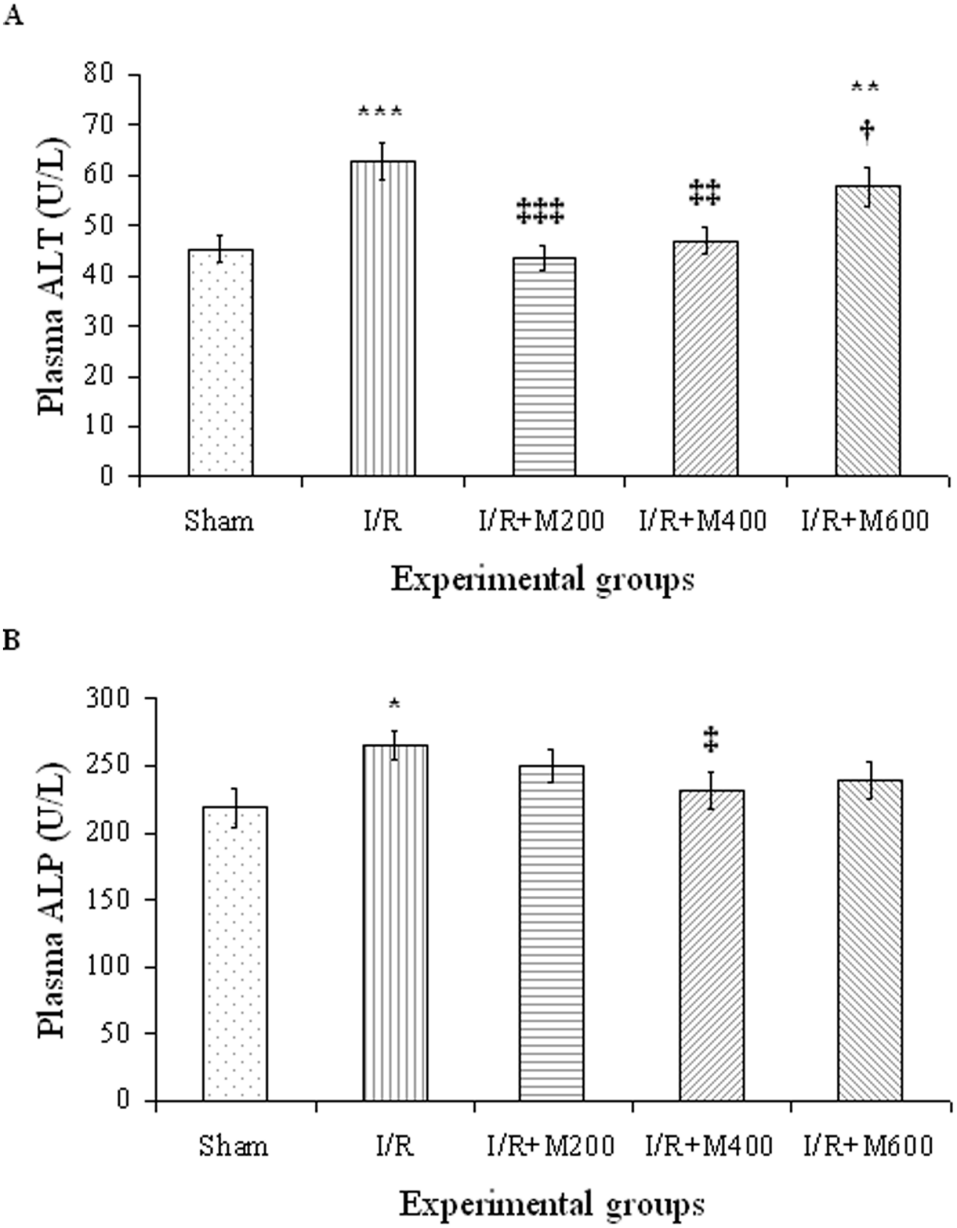
Plasma ALT (A) and ALP (B) levels in rats which underwent renal ischemia/reperfusion and pretreated with normal saline (I/R), or malva silvestris extract at 200, 400, or 600 mg/kg (I/R + M) compared to the sham group. Data is presented as mean ± SE (n = 7). * P<0.05, ** P<0.01, *** P<0.001 in comparison with the sham group ‡ P < 0.05, ‡‡ P < 0.01, ‡‡‡ P < 0.001 in comparison with the I/R group † P < 0.05 in comparison with the I/R + M200 group.

Renal IR led to a significant rise in plasma concentrations of ALP and ALT (Fig 2A and 2B). The rise was more significant for ALT plasma concentrations. Pretreatment with mallow extract at doses 200 and 400 mg/kg led to a complete restoration of ALT (P<0.001 and P<0.01, respectively, compared to the IR group). At the 600 mg/kg dose, however, mallow extract did not restore plasma ALT (Fig 2A). Mallow extract caused significant changes in plasma ALP only at the 400 mg/kg dose (P<0.05) and was ineffective at the other doses (Fig 2B).

### The effect of mallow extract on renal IR-induced inflammation

**Fig 3:**
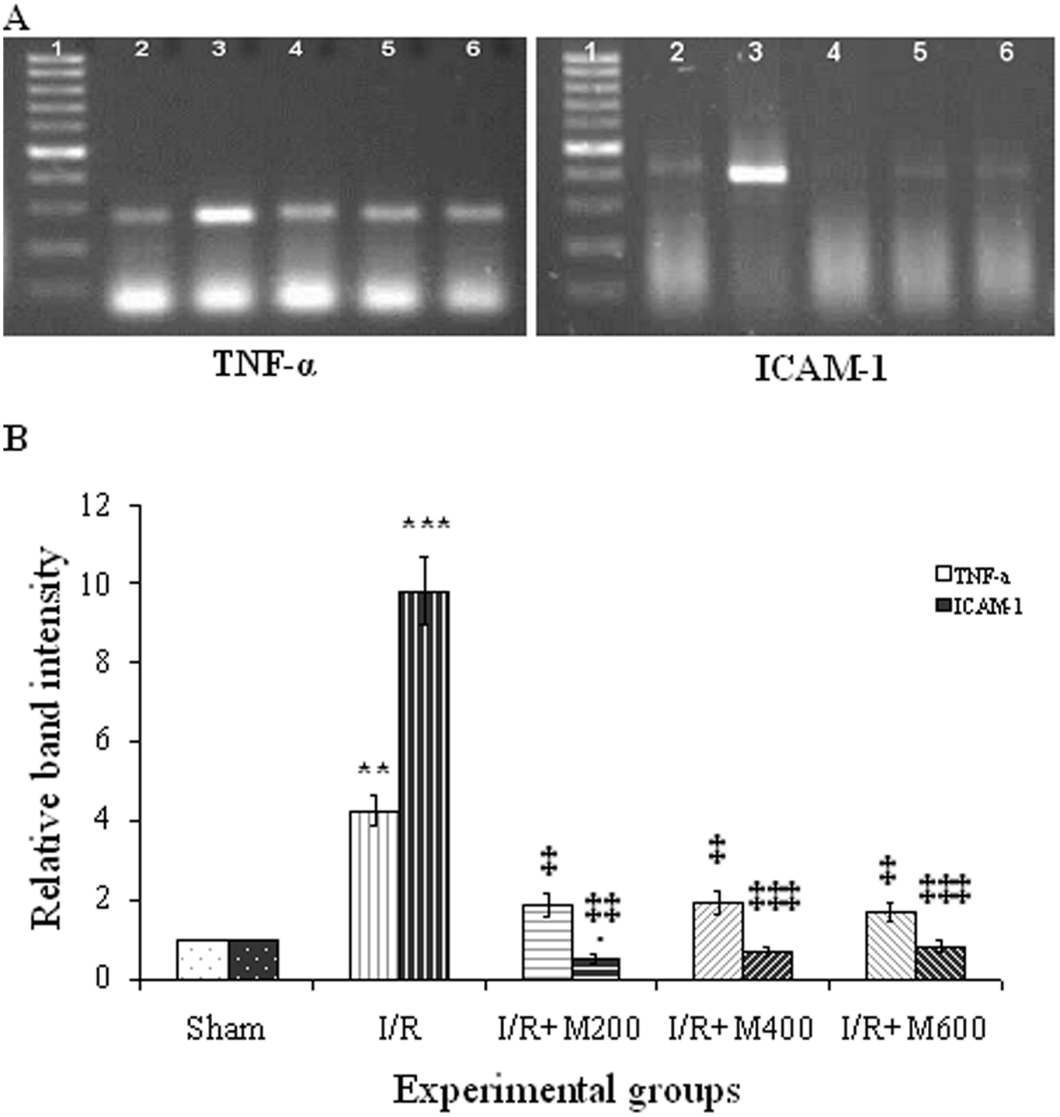
Representative semi-quantitative reverse transcription polymerase chain reaction (RT-PCR) of mRNA encoding tumor necrosis factor-alpha (TNF-α) and intercellular adhesion molecule-1 (ICAM-1) (A) in the renal cortex of the sham group (lane 2) and rats that underwent ischemia/reperfusion after pretreatment with normal saline (lane 3) or malva silvestris extract at 200, 400, or 600 mg/kg (lanes 4–6, respectively). Lane 1 is a 100-bp RNA size marker. Densitometric quantification of relative band intensities from RT-PCR assays for TNF-α and ICAM-1 (B). **P < 0.01; ***P < 0.001 in comparison with their own sham group. ‡P < 0.05; ‡‡‡P < 0.001 in comparison with their own I/R group.

Renal IR led to a rise in the mRNA expression of TNF-α and ICAM-1 in the renal cortical tissue (Fig 3A), and the increase in ICAM-1 was more intense (Fig 3B). Following pretreatment with mallow extract, the mRNA expression of TNF-α was significantly reduced in all the three groups receiving the extract compared to the IR group (P<0.05), reaching the same value as in the sham group. All three doses of mallow extract also reduced the mRNA expression of ICAM-1 significantly (P<0.001).

**Fig 4:**
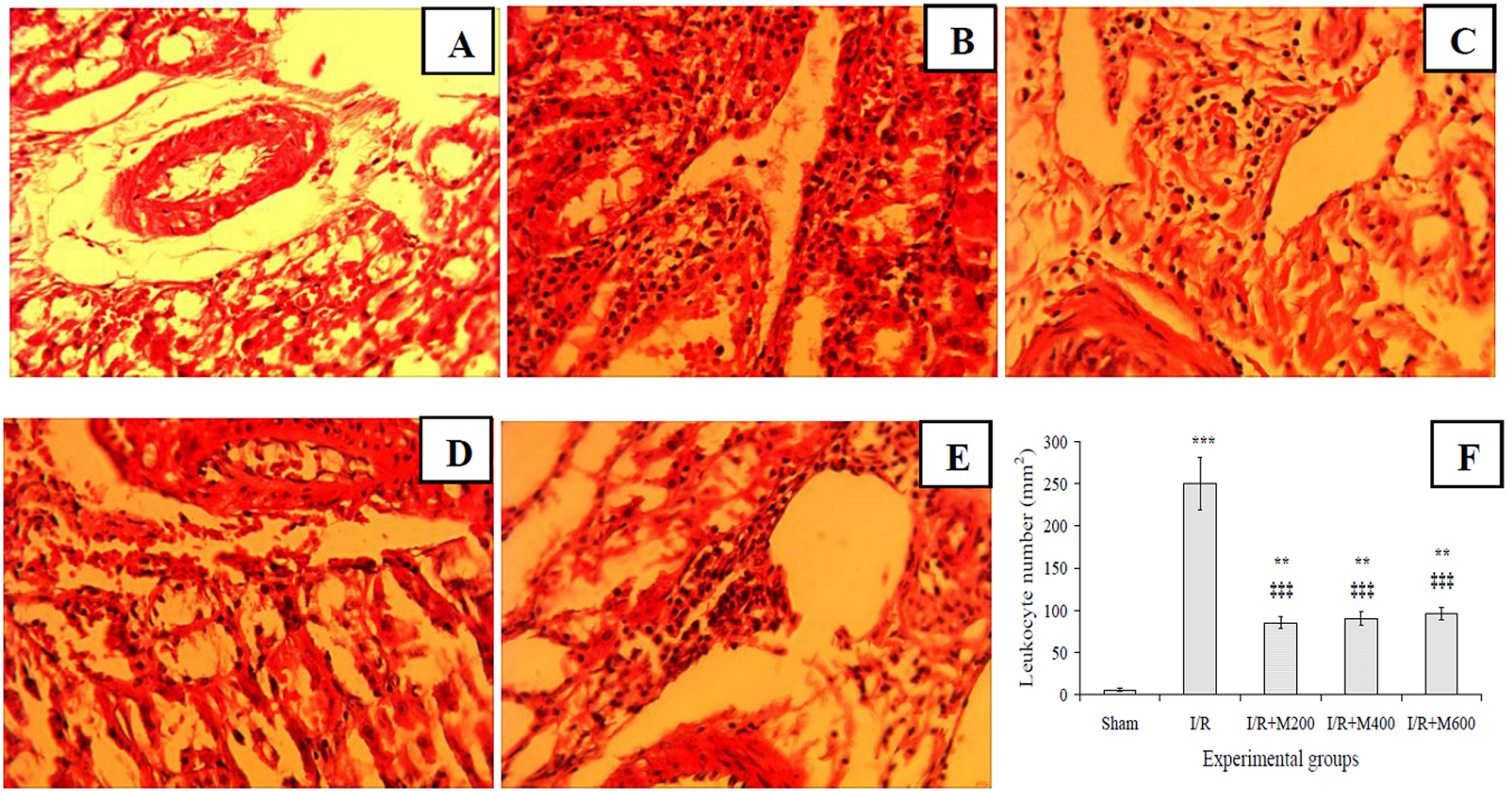
Light microscopic images of renal cortex for representing leukocyte infiltration in rats that underwent sham surgery (A), those that underwent renal ischemia/reperfusion while pretreated with saline (B) or 200, 400, or 600 mg/kg malva silvestris extract (C-E); and leukocyte infiltration as mean ± SE per square millimeters (F). Magnification: 400X.

Furthermore, leukocyte infiltration increased significantly in the IR group compared to the sham group (Fig 4A, 4B and 4F), and all three doses of mallow extract significantly reduced this damage (Fig 4C–4F).

### The effect of mallow extract on renal IR-induced renal and hepatic tissue damage

**Fig 5:**
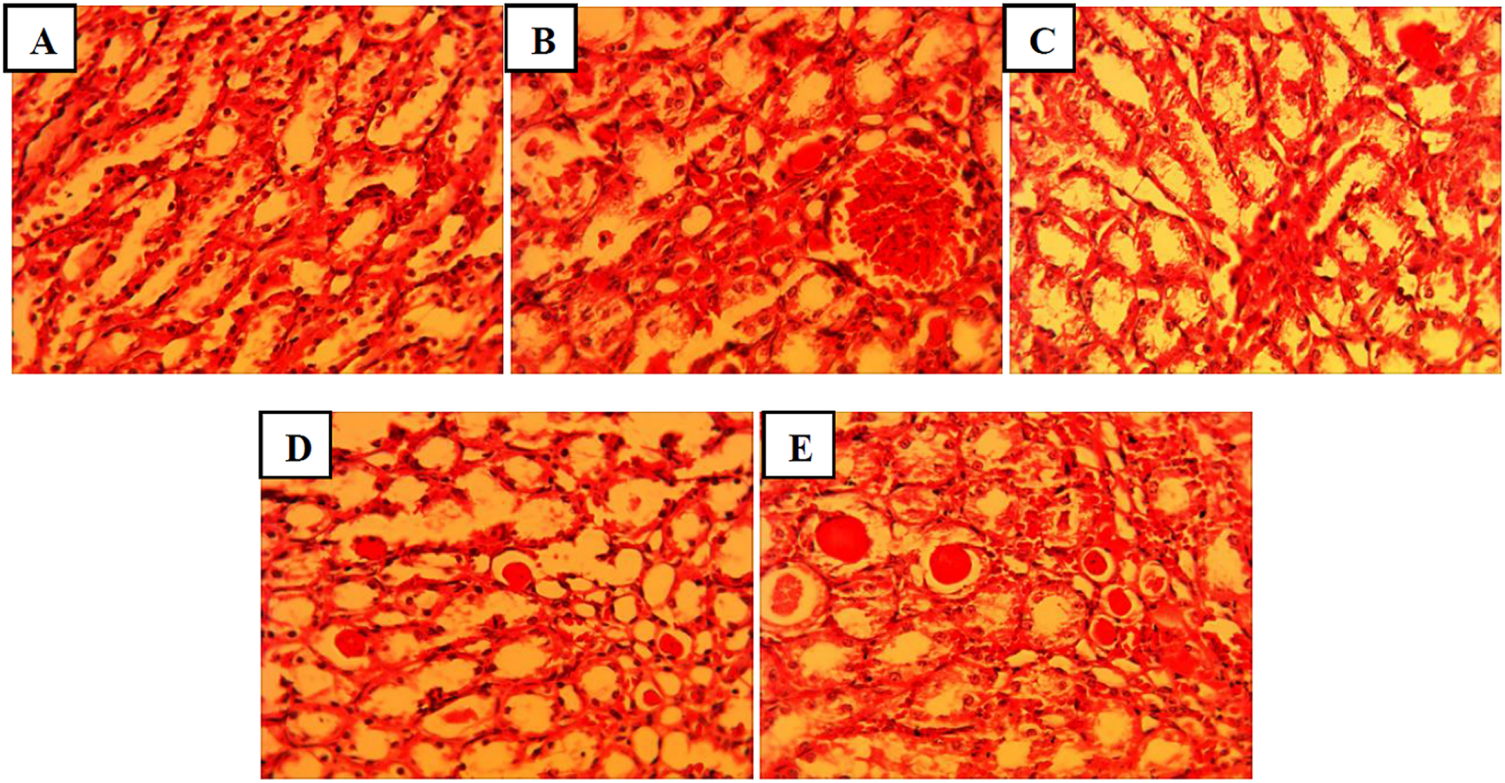
Representing histopathologic alterations in kidneys of rats that underwent sham (A), ischemia/reperfusion and pretreated with normal saline (B), or malva silvestris extract at 200, 400, or 600 mg/kg (C-E). (Haematoxylin-Eosin, 400x).

Fig 5 and Table 2 show that renal IR led to Bowman's space enlargement, intracellular vacuolization, tubular cell necrosis, vascular congestion and intratubular proteinaceous cast; such that the total histopathological score was significantly higher in the IR group compared to in the sham group (P<0.01). All three doses of mallow extract caused a decline in all of these damages, although the rate of decline was higher in the IR+M200 group.

**Table 2:** Renal histological damages induced by bilateral renal I/R, and effect of malva silvestris extract administration on them.

Histopathology	Experimental Groups				
	Sham	I/R	I/R+M200	I/R+M400	I/R+M600
Bowman's space enlargement	0	V	II	II	III
Intracellular vacuolization	0	III	I	II	II
Tubular cell necrosis	0	IV	I	I	II
Vascular congestion	I	IV	II	II	II
Intra-tubular proteinaceous casts	0	V	I	II	III
**Total histopathologic score**	**1**	21**	**7**‡	9*‡	12*‡

Histopathological scores in rats subjected to sham operation and received normal saline (Sham group), ischemia/reperfusion that received normal saline (I/R group), or different doses of malva silvestris (I/R+M groups).

* *P*<0.05,

**P<0.01, in comparison with sham group.

^‡^*P*<0.05, in comparison with I/R group.

**Fig 6:**
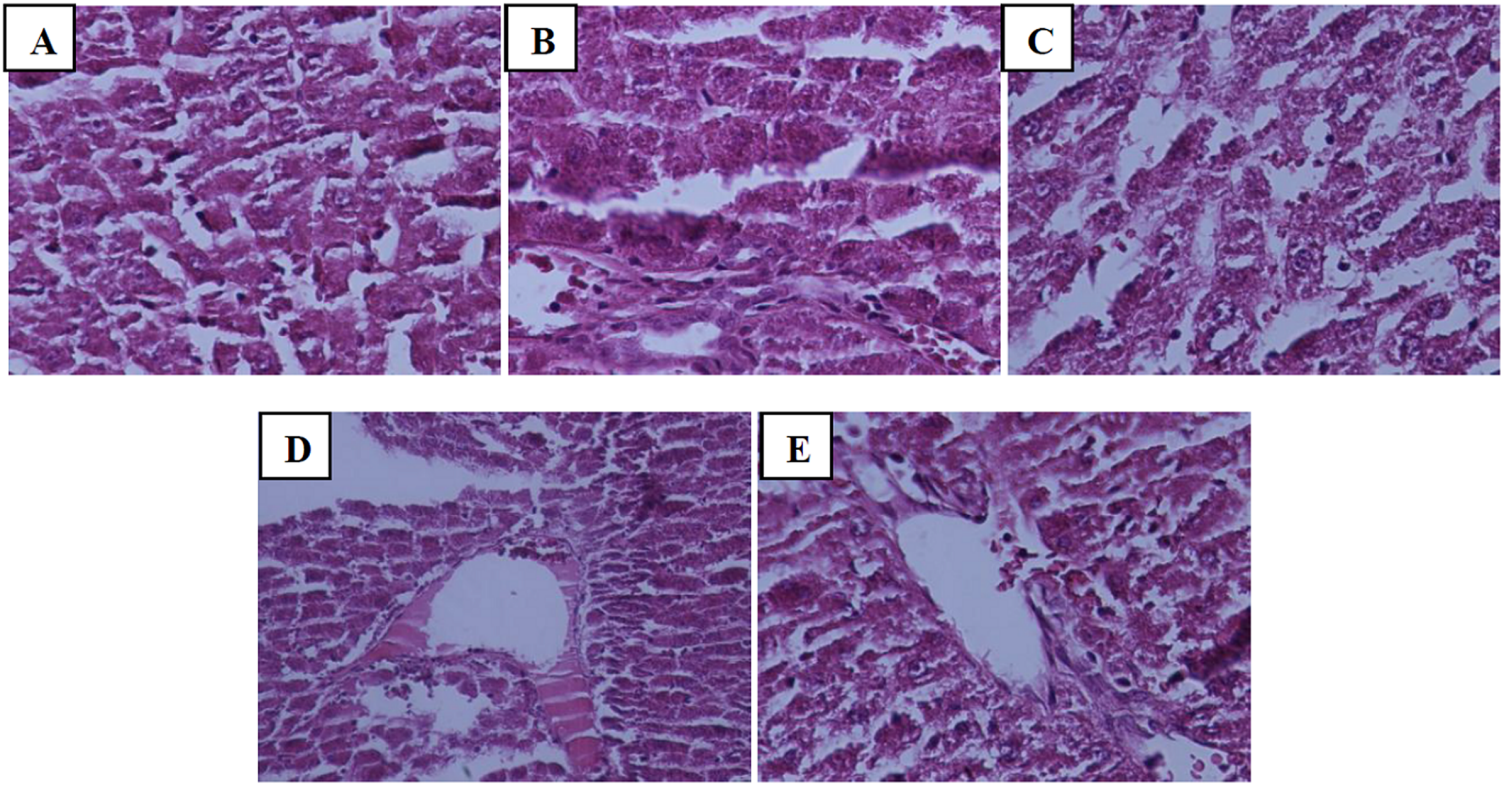
Representing histopathologic alterations in liver tissue of rats that underwent sham (A), renal ischemia/reperfusion and pretreatment with normal saline (B), or malva silvestris extract at 200, 400, or 600 mg/kg (C-E). (Haematoxylin-Eosin, 400x).

Furthermore, renal IR resulted in severe leukocyte infiltration, cell necrosis and congestion in the liver tissue (Fig 6B), while the severity of these damages decreased to moderate degree in the groups receiving mallow extract (Fig 6C, 6D and 6E).

### The effect of mallow extract on renal IR-induced oxidative stress

**Fig 7:**
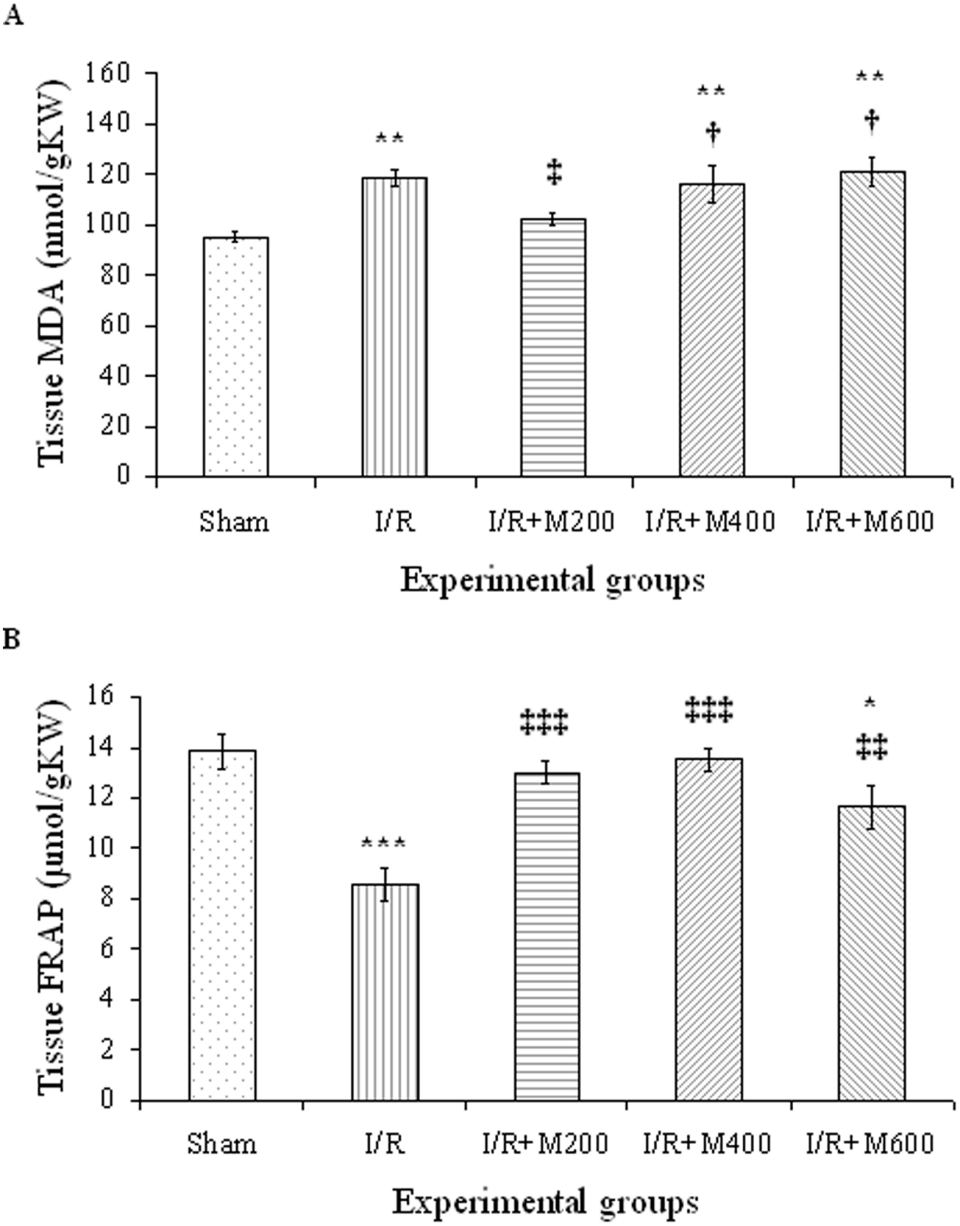
A, Lipid peroxidation level (MDA) and B, total antioxidant capacity (FRAP) in rats that underwent renal ischemia/reperfusion and pretreatment with normal saline (I/R), or malva silvestris extract at 200, 400, or 600 mg/kg (I/R + M) compared to the sham group. Data is presented as mean ± SE (n = 7). * P<0.05, ** P<0.01, *** P<0.001 in comparison with the sham group ‡ P < 0.05, ‡‡ P < 0.01, ‡‡‡ P < 0.001 in comparison with the I/R group † P < 0.05 in comparison with the I/R + M200 group.

The lipid peroxidation level (MDA level) was significantly higher in the IR group compared to in the sham group (P<0.01), and the 200-mg dose of mallow extract reduced it to the same level as in the sham group (P<0.05). However, the 400- and 600-mg doses had no effects on the MDA level (Fig 7A).

The total antioxidant capacity of the kidney tissue was significantly lower in the IR group as compared to the sham group, as indicated by the lower level of FRAP in the IR group (P<0.001). The FRAP level increased significantly in the IR+M200 and IR+M400 groups and reached the same level as in the sham group (P<0.001); however, it was lower in the IR+M600 group compared to the sham group (Fig 7B).

## Discussion

The high mortality rate associated with AKI and its high risk in hospitalized patients necessitate design of strategies for better perception and treatment of this condition. The present study was conducted to examine the protective effects of different doses of hydro-alcoholic mallow extract against renal injuries after 30 minutes of renal ischemia followed by 24 hours of reperfusion, and also against remote organ injuries in the liver.

Many studies have shown that renal IR leads to AKI by causing inflammation, oxidative stress and vascular endothelium and tubular epithelium injuries [24–25]. Cell damage and its resultant molecular products induce inflammation after IR [33]. Necrotic cells release a number of factors into the extracellular space that activate pattern recognition receptors in renal parenchymal cells and dendritic cells (DCs). These activated cells release proinflammatory cytokines [34–36]. Prolonged hypoxia not only causes AKI, but through secretion of profibrotic cytokines it can also lead to chronic kidney disease [35–36]. Renal ischemia also results in the upregulation of adhesion molecules, such as, ICAM-1, P-selectin and E-selectin in the vascular endothelium [37–38], and increases synthesis and release of pro-inflammatory cytokines, such as, IL-1, IL-6 and TNF-α in the kidney [39]. These ischemia-induced inflammatory responses contribute to the development of acute and chronic tissue damage.

In the present study, IR increased expression of the pro-inflammatory factors TNF-α and ICAM-1, and leukocyte infiltration in the interstitial space. Pretreatment with mallow extract significantly reduced the expression of these pro-inflammatory factors and leukocyte infiltration.

Several studies have shown that mallow extract and its ingredients have anti-inflammatory properties [15, 20, 40, 41]. Hydro-alcoholic extract of mallow reduces myeloperoxidase activity and IL-1β level [19]. This anti-inflammatory effect has been in part through a reduction in PGE2 and PGD2 [16], as well as reducing tissue infiltration with neutrophils, and reducing expression of cytokines IL-1β, IL-6 and IL-8 [17–18]. Chemical analysis of the aqueous component of mallow extract has shown that it contains the rutin compound, a flavonoid with anti-inflammatory properties through the selective inhibition of cox-2 [42], and that it inhibitis genes encoding pro-inflammatory factors [43].

The present study shows that mallow extract reduces lipid peroxidation (i.e., reduces MDA) and increases the total antioxidant capacity of the kidney (i.e., increases FRAP).

In the renal IR model, reduced blood flow in the peritubular capillaries leads to tubular hypoxia and stimulates oxidative stress [44]. Moreover, the changes in renal blood flow are not uniform after IR, and as a result, there are hypoxic areas adjacent to areas with normal blood flow. The interaction between these areas increases the production of reactive oxygen species [45–46].

Researchers have also shown that mallow flower extract has antioxidant properties likely owing to polyphenols, vitamin C, vitamin E, beta-carotene, some polysaccharides and essential fatty acids, especially omega-3 and Omega-6 [3, 6, 7]. The antioxidant properties were shown to be directly linked to a phenolic content [47]. Omega-3 has antioxidant and protective properties against the damage caused by renal IR [48]. Mallow also contains enzymes, such as, catalase that destroy H2O2 and protect against oxidative stress [12]. Moreover, a decoction of mallow protected kidneys against vanadium-induced oxidative damage [23].

In our study, renal IR led to a significant AKI as evidenced by increases in plasma creatinine and urea concentrations as compared to the sham group. AKI is characterized by reduced GFR due to increased renal vascular resistance [49–51], resulting in increased plasma concentrations of creatinine and urea. Various factors are reported to increase renal vascular resistance following IR, including activation of tubuloglomerular feedback, sympathetic nervous system, renin-angiotensin system, prostaglandins, platelet-activating factor and endothelial dysfunction [52–53]. Endothelial dysfunction can often be attributed to reduced nitric oxide production. Moreover, TNF-α produced in the kidney following IR reduces blood flow rate and glomerular filtration rate, increases glomerular permeability to albumin and stimulates cell infiltration and ICAM-1 expresion [54].

In our study, the reduced expression of TNF-α after pretreatment with mallow extract might have resulted in restoring the normal blood flow and minimizing AKI.

Various studies have shown that AKI causes liver damage and increases the risk of mortality. AKI is thought to cause liver damage by creating oxidative stress and reducing antioxidant power, increasing pro-inflammatory factors [55], and disrupting activity of cytochrome P450 3A [25]; Thus, antioxidants may help reduce liver damage following AKI [27]. Golab et al. showed that AKI increased liver enzymes, liver TNF-α level, oxidative stress, leukocyte infiltration, apoptosis and cell damage in the liver tissue of rats [56]. However, Gardner et al. [26] showed that in pigs, AKI led only to a transient increase in acute liver injury markers and did not cause apoptosis, edema or cell damage. In the present study, IR-induced AKI increased liver enzymes and led to cell injury and leukocyte infiltration, which is consistent with the results obtained by Golab et al. We measured ALT and ALP as markers of liver damage [57]. The elevation in serum ALT indicated hepatocyte damage. The elevation in serum ALP indicated hepatic biliary damage. In our study, pretreatment with mallow extract significantly reduced liver injury. In line with these findings, Hussein et al. reported that mallow extract protected liver against paracetamol-induced damage, likely through its antioxidant properties [22]. Given that inflammation and oxidative stress are two mechanisms for liver damage after AKI, since mallow extract reduced both it protected liver against remote injuries caused by AKI. The limitation of present study is that we did not obtain baseline measurements of the biomarkers in serum.

## Conclusion

In conclusion, administration of 30-minutes of renal ischemia followed by 24-hours of reperfusion caused significant renal dysfunction, inflammation, oxidative stress, and tissue damage in both kidney and liver. Pretreatment with mallow extract reduced their severity and restored much of these derangements. The hydro-alcoholic extract of mallow was therefore found to have protective effects in the kidney against IR-induced damage, as well as, remote liver damage.

## Abbreviations

ALP: Alkaline Phosphatase
ALT: Alanin Transaminase
FRAP: Ferric Reducing/Antioxidant Power
H & E: Hematoxylin and Eosin
ICAM: Intracellular Adhesion Molecule
MDA: Malondialdehyde
PGD2: Prostaglandin D2
PGE2: Prostaglandin E2
TNF: Tumor Necrosis Factor
